# Handling Variables, *via* Inversion of Partial Least Squares Models for Class-Modelling, to Bring Defective Items to Non-Defective Ones

**DOI:** 10.3389/fchem.2021.681958

**Published:** 2021-07-13

**Authors:** Santiago Ruiz, Luis Antonio Sarabia, María Sagrario Sánchez, María Cruz Ortiz

**Affiliations:** ^1^ Department Matemáticas y Computación, Facultad de Ciencias, Universidad de Burgos, Burgos, Spain; ^2^ Department Química, Facultad de Ciencias, Universidad de Burgos, Burgos, Spain

**Keywords:** process analytical technology, partial least squares, class-modelling, sensitivity/specificity, latent variables model inversion, authentication, attributes

## Abstract

In the context of binary class-modelling techniques, the paper presents the computation in the input space of linear boundaries of a class-model constructed with given values of sensitivity and specificity. This is done by inversion of a decision threshold, set with these values of sensitivity and specificity, in the probabilistic class-models computed by means of PLS-CM (Partial Least Squares for Class-Modelling). The characterization of the boundary hyperplanes, in the latent space (space spanned by the selected latent variables of the fitted PLS model) or in the input space, makes it possible to calculate directions that can be followed to move objects toward the class-model of interest. Different points computed along these directions will show how to modify the input variables (provided they can be manipulated) so that, eventually, a computed ‘object’ would be inside the class-model, in terms of the prediction with the PLS model. When the class of interest is that of “adequate” objects, as for example in some process control or product formulation, the proposed procedure helps in answering the question about how to modify the input variables so that a defective object would be inside the class-model of the adequate (non-defective) ones. This is the situation illustrated with some examples, taken from the literature when modelling the class of adequate objects.

## Introduction

Class-modelling techniques (Forina et al., 2008) focus on the ability of the built class-models for recognizing their own objects (sensitivity of the computed class-model) and rejecting all others (specificity). The additional information that the class-models provide about the categories being modelled, as against a pure discriminant rule, is relevant for authentication of products (Rodionova et al., 2016a), for example, to characterize foods or beverages with recognized quality, such as denomination of origin wines or oil (Barbaste et al., 2002; Marini et al., 2006; Forina et al., 2009; Ruisánchez et al., 2021) combined with spectroscopic and chromatographic techniques to characterize green tea (Casale et al., 2018) with near infrared spectroscopy to antibiotic authentication (Chen et al., 2020) to identify bands for functional spectral data (Hermane et al., 2021) for food-authenticity claims (Oliveri and Downey, 2012) for detection of cold chain breaks in tuna (Reguera et al., 2019), or adulterations (Xu et al., 2013a), or nitro explosive vapors (Pablos et al., 2015). Also, a procedure based on band limits are successfully used as probabilistic one-class classifier (Avohou et al., 2021), among several other applications that can be found in a recent tutorial (Oliveri et al., 2021). In fact, the area is very active: a search in Scopus with key terms “Classification model” OR “Class-modelling” limited to the last five years (2016–2021) and in Chemistry as subject area return 1,013 documents. By reducing the search to (“Classification model” OR “Class-modelling”) AND “Chemometrics”, there were still 431 resulting documents.

The concept of pattern recognition has evolved since the birth of chemometrics (Brereton, 2015) resulting, more than a decade ago, in the classification of the techniques as either discriminant or one-class classifiers (when modelling the categories independently to one another) (Brereton, 2009). A more flexible taxonomy (Rodionova et al., 2016b) distinguishes between “rigorous” (equivalent to one-class classifiers) and “compliant” class-modelling techniques. To build the class-model only objects of the modelled class are considered in the former case while in the latter, objects of different classes are also used. Alternative denominations make distinction between hard or soft models (Brereton, 2011), as those that do not allow or allow overlap between classes, respectively. This division is also used in ref. (Pomerantsev and Rodionova, 2018) for the particular case of PLS-DA (Partial Least Squares Discriminant Analysis (Ståhle and Wold, 1987; Barker and Rayens, 2003), making a distinction between hard and soft PLS-DA models depending on whether they use LDA or QDA (UNEQ) on the PCA-scores of the PLS-predicted responses.

The assumption under the name “one-class classifier” is that each class is modelled independently of any other class, context that covers the situation where in fact there is a single class, e.g., for authentication purposes (Oliveri and Downey, 2012; Rodionova et al., 2016a). In this case, the quality criterion (figure of merit) of the class-model is only its sensitivity, though it can be possible to estimate the specificity as against other samples by using a different set of objects that do not belong to the modelled class (a so-called specificity set in ref. (Forina et al., 2008)). To obtain an unbiased estimate, the specificity set should be representative of all possible “alternative” classes.

Partial Least Squares for Class-Modelling (PLS-CM), first proposed in ref. (Ortiz et al., 1993), is one class-modelling technique that works by implicitly defining probabilistic class-models with predefined values of sensitivity and specificity or, at least, the closest possible to the desired ones with the data at hand. Unlike PLS-DA that also uses a PLS regression model with binary response, PLS-CM first fits probability density functions to the predicted values, separately in each class, which act as probabilistic class-models. For given values of sensitivity or specificity, a decision threshold can be defined as the critical value computed with the fitted distributions. Since the two class-models are fitted together to estimate both sensitivity and specificity, with the distinction in ref. (Rodionova et al., 2016b), the method would be a “compliant” class-modelling method.

Under the same acronym PLSCM, Xu et al. (Xu et al., 2011) build class-models for a single class. The class-model is a kind of confidence interval of the form $\left(1-{\hat{\mu }}_{r}\right)\pm \;z_{1-\alpha /2}{\hat{\sigma }}_{r}$ where ${\hat{\mu }}_{r},{\hat{\sigma }}_{r},$ are estimates computed with Monte Carlo Crossvalidation, of the mean and standard deviation of the residuals of a PLS model with constant response (response matrix **Y** is a vector of ones), assuming that they follow a normal distribution. Accordingly, *z*_1-α/2_ is the critical value of the standard normal distribution for 1—*α* confidence. A new sample is inside the class-model if its predicted response ${\hat{y}}_{un}$ belongs to the interval. Two years later (Xu et al., 2013b), with the new name OCPLS (one-class partial least squares) classifier, the authors add bounds on the allowed variation of the *T* ^2^ statistic as well as on a transformation of the residuals of the regression (difference between one and the predicted responses) to create an outlier identification plot.

Comparing OCPLS (Xu et al., 2013b) with PLSCM (Ortiz et al., 1993), the differences are in the use of one-class (an interval as class-model) or two-classes (probability density functions as class-models) for modelling and that in PLSCM the bounds are imposed as hard constraints in the values of *T* ^2^ and *Q*-residual statistics to reject objects from both class-models.

The membership of an object **x** to a given class-model can be posed as a hypothesis test with null hypothesis H_0_: object **x** belongs to the class-model as against H_1_: it does not. With the usual notation, *α* is the significance level of the test, that is, the probability of type I error (wrongly rejecting the null hypothesis), and *β* is the probability of type II error (fail to reject the null hypothesis). Then, sensitivity of the class-model is 1—*α* and specificity is 1—β, the power of the test. The general notion of type I and type II errors (with probabilities *α* and β, respectively) is usually adapted to the context (Ortiz et al., 2010), and becomes false non-compliance/compliance or false positive/negative, whose meaning is clear once undoubtedly established the hypothesis being tested (the meaning of the “class” we are studying in the class-modelling framework). Speaking in *positive*, the terms sensitivity, specificity, true positive/negative rates, confidence level or power can also be used.

To avoid misunderstanding and facilitate the reading of the paper, in what follows, we will always speak about sensitivity and specificity, which will be estimated as the probabilities that characterize the corresponding class-model, computed with the fitted distributions.

In the illustrative examples in the present work, the class to be modelled is the class of some *adequate* objects, again, understood in a general sense. Besides authentication or fraud detection, another particular situation that fits this framework could be the modelling or monitoring of a process where the class-model of interest is the one for non-defective objects and, clearly, the probability of detecting a defective object (specificity) is important. Furthermore, it can be assumed that the expected failures are known, in other words, that there will be samples representative of the usual defective objects acting as the alternative class. Therefore, the training set for fitting the PLS model has samples representative of both situations: usual defective objects and non-defective ones.

It has been said that with PLSCM, the class-models are defined in the space of the predicted responses. To *backpropagate* them into the input domain requires the inversion of the prediction model. Briefly, the inversion of a model refers to the situation where we have the values of the characteristics we want to achieve (output space), and the aim is to find the values of the predictor variables (input space) to attain them.

The inversion of PLSCM is a LVMI (Latent Variables Model Inversion), term used more frequently in the field of process industry after the seminal papers by Jaeckle and MacGregor (Jaeckle and MacGregor, 1996; Jaeckle and MacGregor, 2000a). A general formulation for LVMI when the latent variables are computed with PLS is in ref. (Tomba et al., 2012) with a through discussion and also a revision of available literature and applications at that time. There, 95% confidence limits on *T* ^2^ and *Q* statistics are already applied to the PLS model fitted with historical data, so that the operating conditions obtained with PLS model inversion must be interior to it. The region defined with these hard constraints on the solutions was later called PLSbox in ref. (Ruiz et al., 2020) where also the explicit consideration of two existing null spaces (one due to the projection into the latent space and the other from the mapping of the scores onto the responses) in PLS model inversion is described. Some more developments about LVMI can be found in refs. (Tomba et al., 2013; Ottavian et al., 2016; Palací-López et al., 2020), and (Zhao et al., 2019a; Zhao et al., 2019b) where the authors propose a modification called the total projection in latent structures of PLS model inversion to take into account that latent variables of a PLS model may contain information irrelevant to the response. Also, by imposing hard constraints on the input domain further to the PLSbox, a different approach to the inversion is in ref. (Ruiz et al., 2018), similar to the one in Lakshminarayanan et al. (Lakshminarayanan et al., 2000) but for inverting PLS2 models. The use of PLS model inversion for product formulation is also noteworthy, especially in the context of Process Analytical Technology with pharmaceutical processes (Tomba et al., 2014; Bano et al., 2017; Palací-López et al., 2019).

In the present work, with PLS-CM, given values of sensitivity and specificity determine a decision threshold *y*_*d*_ to be imposed in the predicted responses, threshold that acts as the boundary of the class-model. The inversion of the built PLS model for *y*_*d*_ would provide values of predictor variables **x**_d_ (a vector in the input domain) whose prediction is exactly *y*_d_.

In general, the solution **x**_d_ is not unique, due to the null space of the PLS model (Jaeckle and MacGregor, 2000b). The null space contains the values of the predictor variables **x**_null_ (vectors in the input space) that are mapped into zero by the linear model, so that any point **x**_d_ + **x**_null_ have the same predicted response *y*_d_.

Since there is a single response (dimension 1), the consideration of the null space when inverting the PLS model would define a (subset inside a) hyperplane in the input space. The objects lying on that hyperplane are at the boundary of the class-model but already in the input space. Moreover, the characterization of this boundary would give indications on how to manipulate or to modify the input variables so that a rejected object can become an accepted one. The details on how to do that are explained in section *Materials and methods*. The computation and possible utility are illustrated in section *Results and discussion* with some data sets taken from the literature. The paper finishes with some conclusions.

## Materials and Methods

### Partial Least Squares for Class-Modelling

Let **X** (*n* × *p*) be a data matrix with *p* variables measured on *n* objects, which belong to two categories, class A and B. This set would be the training set, so that it is assumed that it contains representative samples of these two categories or classes.

The PLSCM method consists of fitting a PLS model to a binary response that codifies the categories. If they are coded as −1 and +1, respectively, the *n*-dimensional vector of responses, **y,** is made up of as many “−1” as objects belonging to category A and as many “+1” as objects of category B in the training set.

The selection of the proper number of latent variables for the PLS model is based on crossvalidation estimates. Throughout the fitting, objects that surpass the 95% confidence limits on both *Q* and *T* ^2^ statistics, if any, are removed and the model is rebuilt.

During the application phase (i.e. when predicting with the fitted model), the predictions are calculated only for the objects with values in both statistics less than the limits stablished (hard constraints, which are *restrictions that determine the envelope of the subspace of acceptable solutions* (Palací-López et al., 2020). Along the paper, to illustrate the methodology, the usual 95% confidence levels are used. Reducing this level would probably shrink the class-models, or the contrary if it is increased, yet in the present work no sensitivity analysis of the results on the confidence levels has been performed.

As PLS models are regression models for fitting quantitative variables, the individual predicted responses ${\hat{y}}_{i}$ are neither −1 nor 1 but different values spreading around −1 and 1. The method then consists on separating these predicted values, according to the class each object belongs to, and probability distributions are fitted independently to each class. Thus, random variable *X*_A_ related to PLS prediction for class A follows a *F*_A_ distribution and *X*_B_, related to class B, follows a *F*_B_ distribution.

Several normality tests are conducted to fit *F*_A_ and *F*_B_. If the normal distribution is not adequate, an alternative distribution will be selected, based on the maximum likelihood.

Without loss of generality, let us suppose that we focus on the class-model of class B (coded as ‘1’). This could be the situation for the particular case of modelling defective/non-defective objects, for example, where class B would be the category of non-defective objects.

In any case, for a given sensitivity *s* in [0, 1], we use the cumulative distribution function of *F*_B_ to compute the critical value *y*_c_ so that $P\left(X_{B}\leq y_{c}\right)=1-s=\alpha$. This critical value will act as a decision threshold, that is, object i-th is assigned to the model of class B when ${\hat{y}}_{i}\geq y_{c}$ and to class A otherwise. Consequently, $y_{c}$ defines the boundary of the class-model. It is worth remembering that alien objects (outside both class-modes) are previously removed with the hard constraints imposed on *Q* and *T* ^2^ statistics.

Finally, the specificity sp of the class-model as against class A is given by $P\left(X_{A}\leq y_{c}\right)$, which is computed with the cumulative distribution function *F*_A_. In this way, as expressed in Table 1 of (Rodionova et al., 2016a) for class-modelling techniques, PLSCM gives a decision rule for a given *α* as a result of the modelling, and sensitivity and specificity can be computed as the usual figures of merit.

**TABLE 1:** Settings of the computed plastic pellets following the direction signaled in Figure 6.

#	Situation	Size5	Size10	Size15	TGA	DSC	TMA
1	Rejected	14.24	10.07	34.43	622.00	18.73	52.08
2	Rejected	13.51	8.91	32.15	638.47	18.67	53.60
3	Rejected	13.03	8.13	30.63	649.45	18.63	54.61
4	Accepted	12.66	7.55	29.49	657.69	18.60	55.37
5	Accepted	12.29	6.96	28.35	665.92	18.56	56.13
6	Accepted	11.93	6.38	27.21	674.16	18.53	56.89
7	Accepted	11.56	5.80	26.07	682.39	18.50	57.65
8	Accepted	11.20	5.22	24.93	690.63	18.47	58.42
9	Accepted	10.83	4.64	23.79	698.87	18.44	59.18
10	Accepted	10.47	4.05	22.65	707.10	18.41	59.94
11	Accepted	10.10	3.47	21.51	715.34	18.38	60.70

### Inversion of a Partial Least Squares Model

Once fitted a PLS model, its typical use is to predict values of *y* given **x** (*p*-dimensional vector of predictor variables). The reverse situation, looking for the values of **x** whose prediction is a predefined *y* requires the inversion of the regression model.

In the context of process control or product formulation with a PLS prediction model, its *direct* use means predicting quality characteristics of the product manufactured with given settings **x** of input variables (process variables, characteristics of material including their amounts mixed, environmental variables, etc.). Thus, the inversion of the PLS model would refer to the situation where we have the desired characteristics and need to find the settings of the input variables, if any, to attain them.

In the following, we will introduce the inversion of the PLS model for a single response, which is the only situation that applies here. With the notation stablished in the previous section, **X** (*n* × *p*) is the matrix of predictor variables and **y** is the response vector with the *n* binary values. In the class-modelling situation, the PLS model fitted to **X**-**y** leads to defining different threshold values *y*_d_, each one related to a pair (sensitivity, specificity) that qualifies the corresponding class-model.

Consequently, by defining *y*_*d*_ as the target value, the inversion of a PLS-CM model would provide values of the predictor (input) variables that are mapped exactly into *y*_d_ via the PLS model, i.e., the characteristics of the objects that are directly projected into the class-model boundary. Therefore, setting aside the uncertainty in the prediction of any data-driven model, these objects would represent the boundary of the class-model already in the input space. Since PLS is a linear model, the boundary thus constructed is also linear. These ideas are developed in a more precise way in the following lines.

With a single response in the response space, like in this case, the inversion of the PLS model with *a* latent variables can be computed algebraically because it consists on solving Eq. 1 in **x**.

$$\hat{y}=TQ^{T}=x^{T}WQ^{T}$$ (1)

where **T** (*n* × *a*) is the matrix of common scores, **W** (*p* × *a*) is the weights matrix and **Q** (1 × *a*) is the **y**-loadings matrix (which is a row vector in this case). As usual, superscript *T* means transposing.

The input space of predictor variables has dimension *p* and the dimension of the output (response) space is one. Therefore (Lay et al., 2016), the kernel of the PLS model (null space of **QW** ^*T*^), which is the set of points with null response, has dimension *p*—1 > 0 unless *p* = 1, which would be a very unrealistic situation. Therefore, the null space is a hyperplane in the input space passing through zero (*p-*dimensional vector of null coordinates), that is, a linear subspace.

Because of their own definition, any vector in the null space adds variability in the input space without modifying the predicted value. That means that, given a desired *y*_d_, for any *p*-dimensional solution of the inversion, that is, any vector **x**_d_ with $x_{d}^{T}WQ^{T}=y_{d}$ , all the remaining solutions of Eq. 1 can be written as

$$\left\{x_{d}+x_{0}\;:x_{0}^{T}WQ^{T}=0\right\}$$ (2)

Hence, the inversion has infinitely many solutions for *y*_d_, although it suffices to consider one of them and characterize the null space.

A sequential alternative for the inversion starts by finding the vector of scores **t**_d_ (*a*-dimensional) such that

$$y_{d}=t_{d}^{T}Q^{T}$$ (3)

In this sequential approach, the dimension of the latent space spanned by **T** is *a* so the null space inside the latent space has dimension *a*—1 (which is positive for more than one latent variable), i.e., for *a* > 1 the null space is also a hyperplane, but inside the latent space.

Because of this null space, the solution of Eq. 3 is not unique either, there are infinitely many solutions described from any particular one, **t**_d_, in the set in Eq. 4.

$$\left\{t_{d}+t_{0}\;:t_{0}^{T}Q^{T}=0\right\}$$ (4)

All *a*-dimensional vectors belonging to the set in Eq. 4, solutions of Eq. 3, lie on a hyperplane in the *a*-dimensional latent space that, contrary to the null space, does not contain the null vector (unless, of course, *y*_*d*_ = 0).

This property about null spaces of linear models has been already used in ref. (Largoni et al., 2015). to divide the latent space into two subspaces, one for on-spec batches and the other for off-spec batches, depending on an end-point product quality.

In the present context with PLSCM, given the threshold value *y*_d_, the hyperplane in Eq. 4 is in fact the decision boundary of the class-model in the latent space. Moreover, via the **X**-loadings matrix **P** (*p×a*), Eq. 5 gives the objects in the input space whose projection are the scores in Eq. 4.

$${\hat{x}}_{d}=\left(t_{d}+t_{0}\right)P^{T}\text{with}\;t_{d}^{T}Q^{T}=y_{d},\text{and}\;t_{0}^{T}Q^{T}=0$$ (5)

Because all the scores in Eq. 4 lie on the same hyperplane, the corresponding input objects computed with Eq. 5 also belong to a subspace of dimension *a*−1 inside the *p*-dimensional input space.

However, once in the input space and if *p* > *a* (which is usually the case), there are still some more solutions of the inversion, additional to the ones computed with Eq. 5. They correspond to a (*p—a*)-dimensional subspace obtained when adding points (*p*-dimensional vectors) that belong to what we have called the **W**-null space (Ruiz et al., 2020), spanned by the loadings of the latent variables discarded when building the PLS model.

Consequently, the solutions in **x** of Eq. 1 for $\hat{y}=y_{d}$, described in Eq. 2, are also described as in Eq. 6, where ${\hat{x}}_{\text{d}}$ is defined in Eq. 5.

$$\left\{{\hat{x}}_{d}+x_{\text{w}0}\;:x_{\text{w}0}^{T}W=0\right\}$$ (6)

A final consideration is worth mentioning. Although the PLS prediction for all the points in either Eq. 2 or Eq. 6 will be *y*_d_, not all of them define a *feasible* object or, in general, a valid solution of the inversion. The valid solutions are those that belong to the PLSbox (Ruiz et al., 2020), which is the region of applicability of the model, characterized by the limits imposed on both the *Q* and *T* ^2^statistics when fitting the PLS model; and that also belong to a given domain *D* inside the input space, that accounts for the characteristics of the input variables in each particular application. This domain should be explicitly defined since it imposes additional hard constraints for the valid solutions of the inversion.

For the present work, the PLSbox is defined with the limits at 95% confidence level. The domain *D* on its part is defined with the range of the variables in the training set, which at least describes the physical bounds on the predictor input variables (Tomba et al., 2012).

In what follows, we will only consider *valid* (*feasible*) solutions of the inversion, that is, points whose prediction is *y*_d_ and that belong to both *D* and the PLSbox.

If the situation were one that fits any form of process control, or product formulation, the general principle in model inversion problems is to manipulate the variables that can be manipulated (in a process control sense or compositional variables) to obtain a product as close as possible to the required specification (Dunn, 2020).

The *specification* in the situation being discussed is related to sensitivity and specificity of the class-model, and the solutions of the inversion give the boundary of the class-model. Thus, different directions of *manipulation* (of scores inside the latent space or of variables in the input space) can be defined, any of them crossing the boundary at some point so that following the direction allows moving in or out of the class-model.

In the latent space, the most easily computable direction is the one defined by the normal vector of the boundary hyperplane (i.e., the vector perpendicular to the hyperplane) which is **Q** ^*T*^. This direction does not depend on the inversion of the model but the precise position of the hyperplane does, that is, at least one solution of the inversion is needed to have the boundary that allows deciding whether a given object is inside or outside the class-model.

The same idea can be applied directly in the domain *D* to define a direction of movement/manipulation of the input variables. In this case, it would be the straight line whose director vector is **QW** ^*T*^, orthogonal to the global null space of the fitted PLS model and, thus, to any hyperplane computed as in Eq. 2 or Eq. 6, that positions the boundary of the class-model in the input space.

### Data Sets

Two different data sets are considered to illustrate the proposed method. The first one does not come from a process with attributes data but illustrate other situations, provide some of the variables can be manipulated. The second one will emulate the use of historical data to fit a model that helps in process control and/or product formulation.

The first data set^a^ contains samples of 128 red young wines from Spanish DOC (*Denominación de Origen Calificada*) Rioja (Ortiz et al., 1995). The wines are characterized by six variables related to physical-chemical measures of color, namely red/green chromaticity (*a*), yellow/blue chromaticity (*b*), lightness (*L*), chroma (*C*), hue (*H*), and saturation (*S*). Expert tasters visually assess the color of each wine and divide the objects into two categories, acceptable or non-acceptable wines because of their color.

The second data set^b^ contains six characterizing measurements for batches of plastic pellets, which will be the predictor input variables, with 24 rows. The first three characteristics, coded for confidentiality, are related to the percentage material in the mixture with different size range (size5, size10 and size15). The last three characteristics are measurements from TGA (thermal gravimetric analysis), DSC (differential scanning calorimetry) and TMA (thermomechanical analysis) devices. The outcome when using this material is either Poor or Adequate.

## Results and Discussion

### Rioja Red Wines

Predictor matrix **X** is 128 × 6 and response **y** is a vector with binary values, namely −1 for non-acceptable wines and one for the acceptable ones. With autoscaled **X** and **y** and leave-one-out crossvalidation, a three latent variables PLS-model is fitted that explains 91.01% of variance in **X** with 72.86% in **y** (70.88% in crossvalidation).

The predicted responses corresponding to non-acceptable wines are fitted to a normal distribution with mean -0.95 and standard deviation 0.45 (the smallest *p*-value among the tests performed was greater than or equal to 0.10, thus, the idea that the values come from a normal distribution cannot be rejected with 90% or greater confidence). On the contrary, the responses corresponding to acceptable wines are not compatible with a normal distribution. The minimum log likelihood was similar for a beta distribution with four parameters and to a highly asymmetric triangular distribution with three. This was the one selected with lower limit −0.57, center point 1.16 and upper limit 1.17.

Without loss of generality, let us focus in the class of acceptable wines, codified as 1. The fitted probability distributions allow setting different decision thresholds *y*_*d*_ which, in turn, are related to different values of sensitivity and specificity for the class-model of the acceptable wines.

**FIGURE 1:**
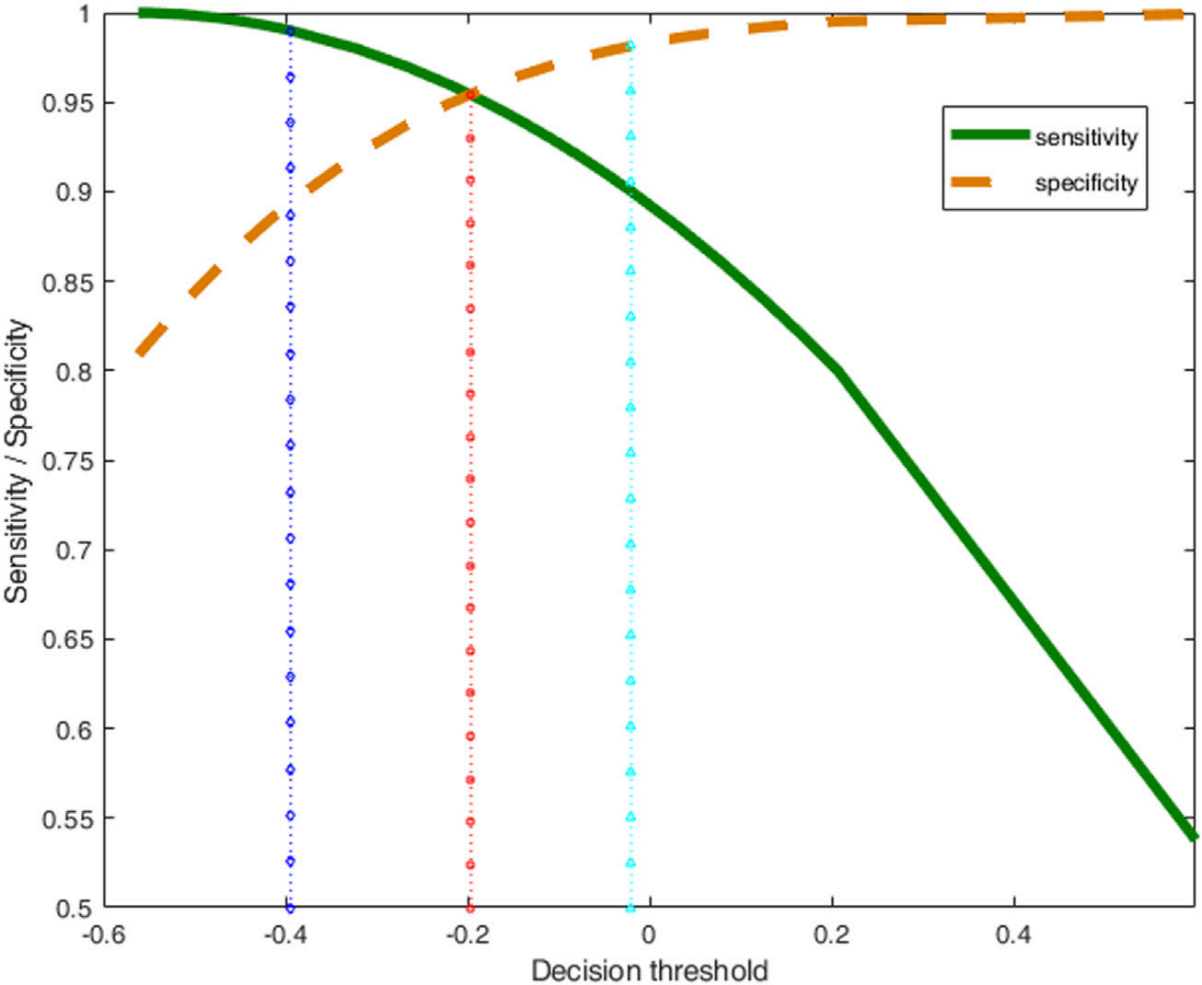
Sensitivity (green thick line) and specificity (brown dashed line) of the class-model of ‘acceptable’ wines as a function of the decision threshold. The dotted vertical lines mark the decision thresholds for obtaining different class-models in terms of their sensitivity and specificity.

These values are depicted in Figure 1 (green continuous line for sensitivity, brown dashed line for specificity) as a function of the decision threshold. It is clear how larger threshold values results in an increase of specificity (dashed line), linked to a decrease of sensitivity (continuous line).

From the set of possible class-models computed with PLS-CM, the more balanced one is the one indicated with the vertical red dotted line, with little squares in Figure 1, for which we expect the same values of sensitivity and specificity, 0.954 in this case, that corresponds to *y*_d_ = −0.196.

**FIGURE 2:**
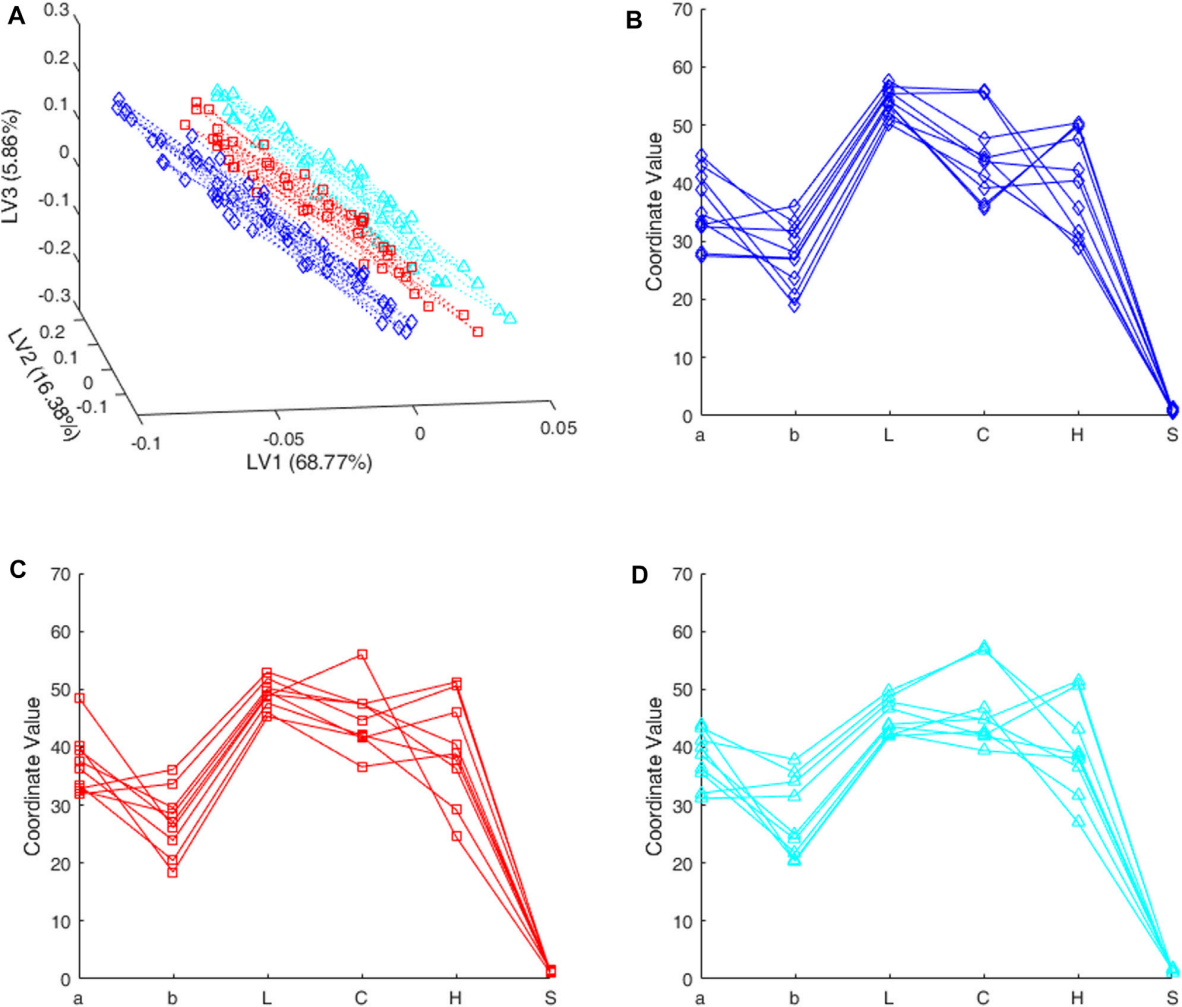
Rioja wines. Boundary objects for different class-models **(A)** latent space **(B–D)** input space. Blue lines and rhombuses are for the class-model with sensitivity 0.99 and 0.89 of specificity; red lines and squares are for the balanced class-model with both sensitivity and specificity equal to 0.954; cyan lines and triangles are for the class-model with sensitivity 0.90 and 0.98 of specificity.

By using this *y*_*d*_ as target value, the inversion of the PLS model would provide points in the input space (where the objects vary) whose predicted response will be exactly the decision threshold *y*_d_, according to Eq. 1 with $\hat{y}=y_{d}=-0.196$. Working sequentially, the solutions of Eq. 3 are scores in the latent space, some of them depicted in Figure 2A as red squares.

By using the loadings as in Eq. 5, the corresponding points in the input space are in six dimensions. Therefore, the usual Cartesian representation is not available. Extensions to visualize data in greater dimension includes the so-called matrix plot, which consists of a set of two-by-two Cartesian plots for any two variables. This matrix plot is usually more informative when representing the scores of a PCA (Principal Component Analysis) that better describe the internal correlation structure of data.

Another alternative, whatever we are visualizing, the Parallel Coordinates Plot also helps in describing the joint behavior of the variables (the “coordinates” of the points). The value of each coordinate is plotted as height above the ordinate axis, against its position in the vector. Then, the values are linked together by a broken line to follow each point. Therefore, rather than its usual meaning, the abscissa axis only accommodates as many slots as coordinates in the point. Although with this disposition there is no limit to the dimension of the points depicted as Parallel Coordinates Plot, it becomes messier when increasing the number of coordinates.

In any case, the points in the input space that correspond to the red squares in Figure 2A are depicted also in red in Figure 2C in the form of a parallel coordinates plot. In both cases, we are seeing points falling on the boundary of the class-model, whether scores in Figure 2A or raw variables in Figure 2C.

If the requirements on the class-model change, the decision threshold *y*_d_ also changes. To illustrate this property, the inversion procedure is repeated for another two different threshold values, in blue and cyan vertical lines in Figure 1, that correspond to the class-models with the usual 0.99 and 0.90 sensitivity, respectively.

Figure 2 also shows some new valid solutions predicting every threshold in both the input and latent spaces. Figure 2B and Figure 2D depict the raw variables in the input space (in the domain *D* defined by the range in **X**) in the form of a parallel coordinates plot. Figure 2A is the plot of their projection (scores) in the 3D-latent space. In both cases, the solutions in blue (lines and rhombuses) are for the class-model with sensitivity 0.99 (with 0.894 of specificity, see Figure 1); cyan lines and triangles are for the class-model with sensitivity 0.90 (specificity 0.981).

As we have a single response, the null space in the latent space is a plane because we have three latent variables. Consequently, the projection of the computed solutions into the latent space will be in the corresponding 2-dimensional subspace. The dotted lines in Figure 2A are meant to help observing how the points of the same color lie on the same plane, and different colors and symbols define different parallel planes in the latent space.

It is less clear but the corresponding objects in the **X**-space in Figures 2B–D are in a two-dimensional subspace inside the boundary of the different class-models, and thus they correspond to some kind of prototype discriminating objects. To make graphs clearer, only around fifty points were calculated for each threshold. However, any convex combination of any pair of points in Figure 2 is also a valid solution and therefore belongs to the boundary of the class-model at hand.

In any case, the solutions depicted have different values for the variables, in particular, we see how the boundary objects for the balanced class-model in red, that clearly occupy an intermediate position among scores in Figure 2A, have not so clear differences in Figure 2C, when comparing with Figures 2B,D.

Finally, there are some more possibilities that do not come from the latent space or, in other words, that predict the same threshold value but are projected into the origin of the latent space. All points together, added to a particular solution as in Eq. 6, define the boundary of the class-model (a hyperplane) in the domain *D* of the input variables.

From the practical point of view, it is probably more interesting to notice that the probability of being inside the class-model of accepted wines increases when moving in the latent space, graphically in Figure 2A, from scores near the blue rhombuses (which, in fact, define a plane), traversing the red squares toward scores ‘above’ the cyan triangles which define another plane.

Obviously, each wine is projected into a unique position in the latent space and its acceptance or rejection depends on the sensitivity and specificity selected to make the decision. However, for a given class-model, we can compute scores (ideal scores not necessarily corresponding to any of the wines in the training set) moving in the direction of improving the color toward the acceptance of the wine.

For example, let us consider the balanced class-model (in red lines or squares in Figure 2 with sensitivity and specificity both equal to 0.954) and let us take one of the wines rejected with the class-model, **x**_d_, which is outside the class-model of the acceptable wines, with a 0.046 probability (4.6%) of being wrongly rejected.

**FIGURE 3:**
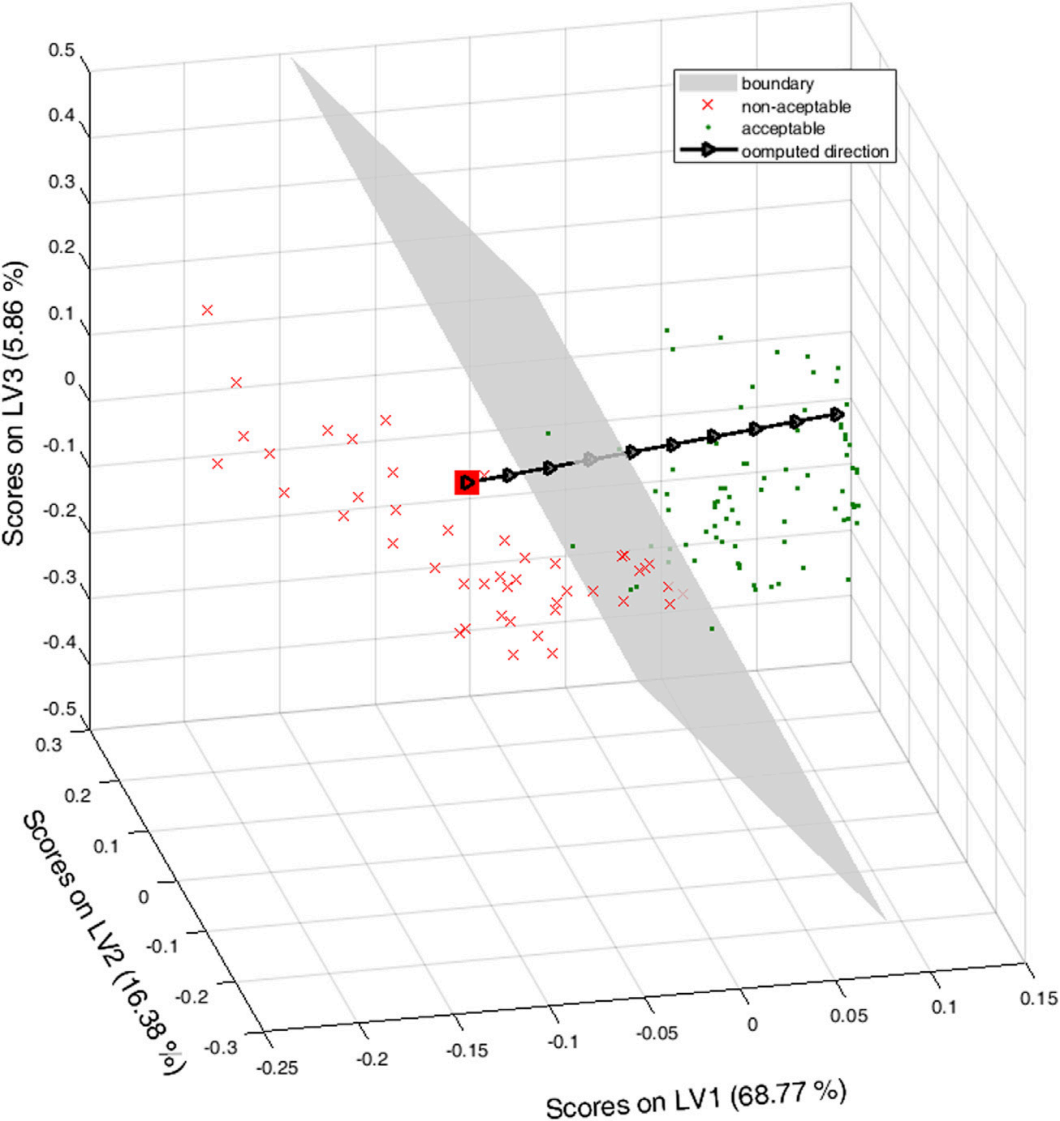
Latent space for the Rioja wines. Green points are for acceptable wines, red crosses and the red filled square for non-acceptable ones. The grey plane is the boundary plane for the class-model when sensitivity and specificity both equal to 0.954. The black triangles are along the direction of improving the color of the wine.

Its projection into the latent space is the filled red square in Figure 3, where the boundary plane is depicted in grey extending the convex hull of the red squares in Figure 2A to better illustrate the indeterminacy due to the null space. For reference, the scores of the training set are also depicted, red crosses for the non-acceptable wines, green points for the acceptable ones.

Filled black arrows in the black line in Figure 3) mark an ideal direction of improving the color, discretized by taking 10 points equally spaced along the line segment orthogonal to the plane and starting in **x**_d_. Graphically, it is clear that, at some point, the computed score crosses the plane and then, the corresponding object would be inside the class-model of acceptable wines.

**FIGURE 4:**
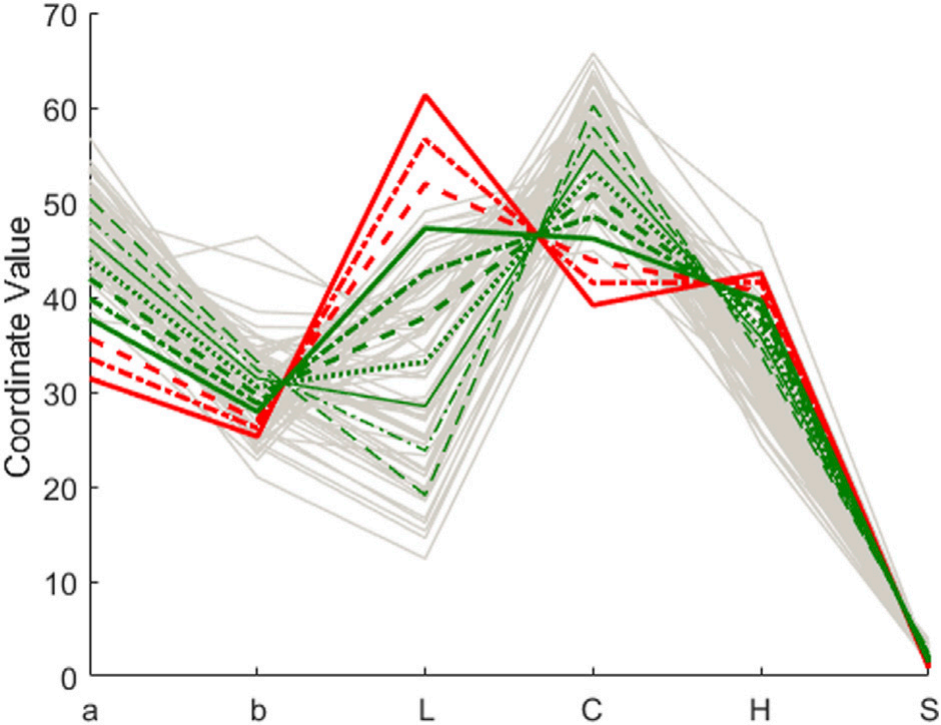
Rioja wines. Parallel Coordinates Plot for objects in the input space. In grey the wines inside the class-model with sensitivity and specificity 0.954. The red continuous line is **x**_d_, the remaining colored lines are for the points computed, rejected in red, and accepted in green.

The objects in the input space whose projections are the ten scores along the black line in Figure 3 are the colored lines in the Parallel Coordinates Plot in Figure 4, from the continuous red line (that corresponds to the non-acceptable wine **x**_d_) to the dash-dotted and dashed red lines, both still for rejected objects.

Following further the same direction pointed in Figure 3, we have the continuous green line, already inside the class-model and the remaining green lines (dot dashed, dashed, dotted and thinner continuous, dot-dashed and dashed green lines) depicting objects that would be “more and more clearly” inside the defined class-model and, hence, accepted. For reference, the light grey lines in Figure 4 are the wines of the training set accepted with the class-model. It is clear that the green lines are, more and more, among the real values of the acceptable wines.

We have already said that, except for the red continuous line, the remaining colored lines in Figure 4 are computed points. Nevertheless, they show how the movement along the line in Figure 3 is related to a systematic variation of the input variables. Following the different lines in Figure 4, we see that to improve the color of the wine toward its acceptance, it is necessary to increase *a* and (to a lesser extent) *b*, decrease *L*, increase also *C*, decrease *H* and slightly increase *S*, but always maintaining the exact relation (relative systematic variation) shown in Figure 4.

Although there is more than one direction to exert the same effect, with the one selected, it is clear that moving the colorimetric parameters in the adequate range and relation, which is viable for an expert oenologist by mixing different wines, it is possible to get closer to and eventually inside the class-model of acceptable wines, based on their color.

### Plastic Pellets

In this case, matrix **X** of predictor variables is 24 x 6. The outcome when using the corresponding material, either poor or adequate, is coded into −1 and 1, respectively, to form the matrix of binary responses to be predicted.

With autoscaled predictors in **X** and binary responses in **y**, also autoscaled, a PLS model is fitted with two latent variables that explain 73.64% of the variance in **X** and 66.17% of the variance in **y**, with $R_{cv}^{2}$ = 56.65% (obtained with venetian blinds, ten data splits, one sample per blind).

The low predictive ability of the model could be due to the small number of samples at our disposal. This implies that the conclusions obtained can carry great uncertainty, which is one the reasons why the results should be experimentally validated, whenever possible. However, the example is still valid to show how to proceed.

The PLS-predictions for the class adequate are fitted to a *N*(0.42, 0.48), with the smallest *p*-value for several normality hypothesis tests being greater than 0.10. The small number of samples in the class poor prevent testing the normality, though the points are well aligned in the ‘normal probability plot’. Therefore, for the computation of sensitivity and specificity the *N* (−1.01, 0.49) is used for the poor class.

**FIGURE 5:**
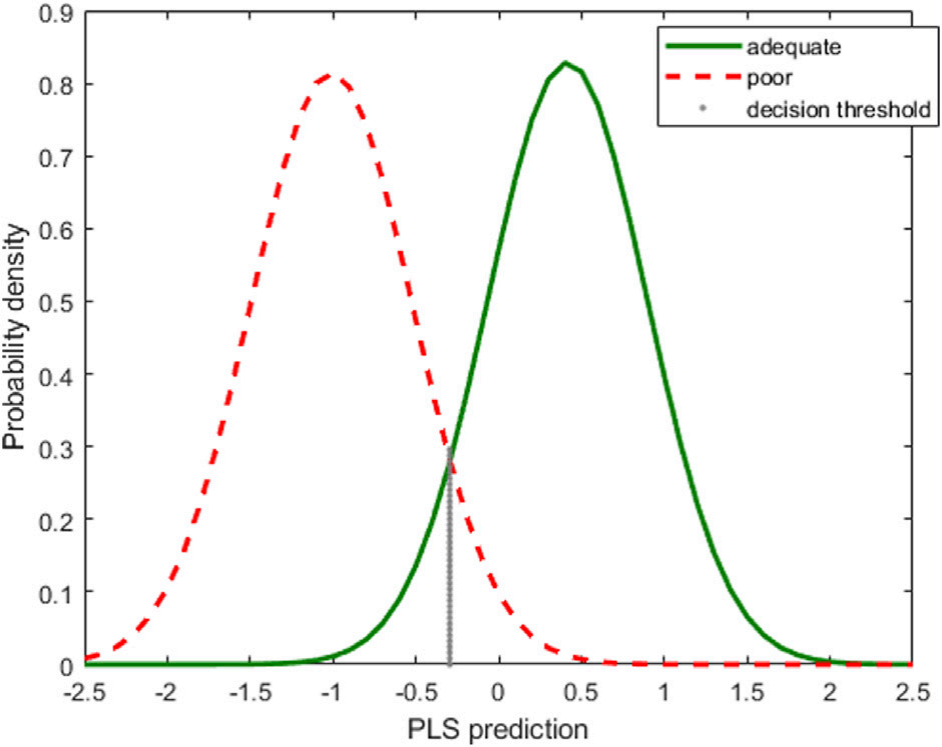
Plastic pellets. Probability density functions of the normal distributions fitted to the PLS predictions, red and dashed for the poor category, green for the adequate one. The vertical dotted line marks the decision threshold for equal sensitivity and specificity.

The corresponding probability density functions of the fitted distributions are depicted in Figure 5, red dashed line for the poor category, green continuous line for the adequate one. Again, we focus on the class of adequate pellets, coded as 1, that mimics the situation of a process control with attributes data: one minus the sensitivity of the class-model would be the probability of false alarm and the specificity would be the power to detect a true defective (poor) object.

Choosing a threshold value for PLS predictions, for instance the one marked with the vertical dotted line in Figure 5, means defining a class-model whose sensitivity is the probability under the green curve to the right of the line, whereas the specificity would be the probability under the red dashed curve to the left of the black vertical dotted line.

In fact, usually, first the sensitivity and specificity required for the decision are set, and then, taking into account the fitted distributions, the decision limit *y*_d_ is computed. In the illustration of Figure 5, the value *y*_*d*_ = -0.2913 corresponds to the class-model with the same sensitivity as specificity, namely 92.9%.

**FIGURE 6:**
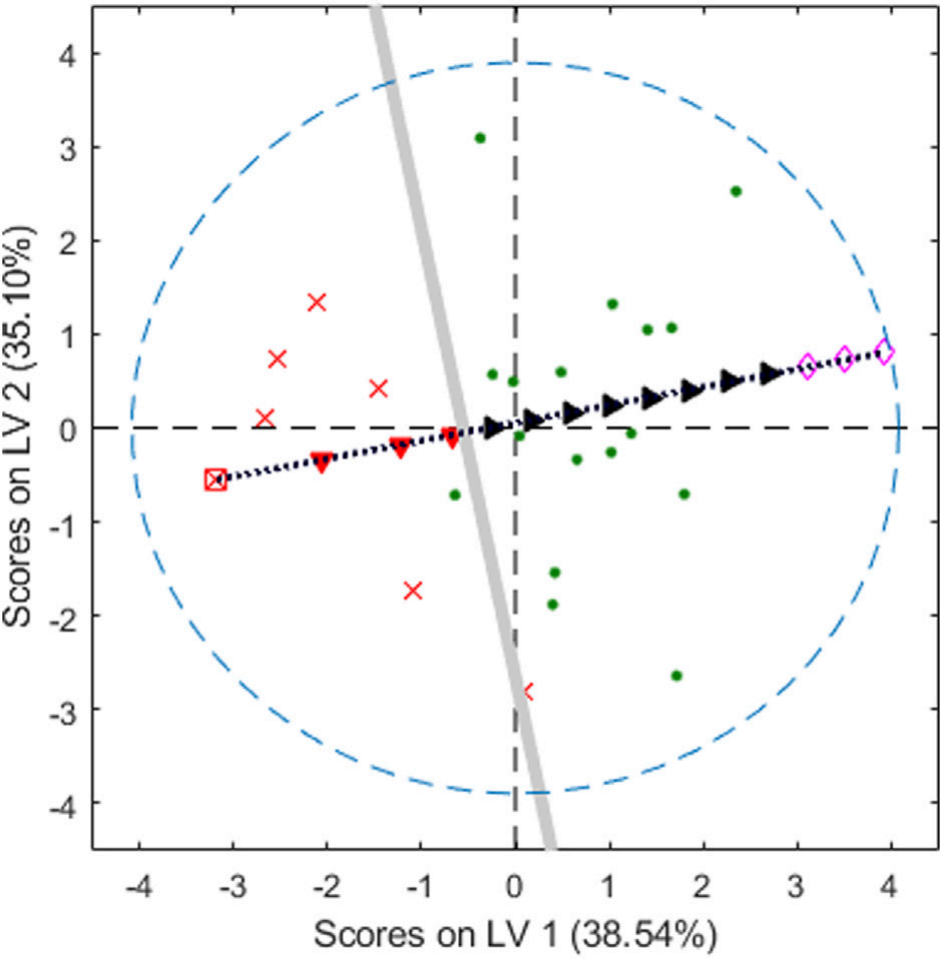
Plastic pellets. Second vs. first latent variables. Green points for adequate objects, red crosses, and the square, for the poor ones. The thicker grey line is the decision line with 93% sensitivity and specificity and the black dotted line is the direction of ‘repairing’ the *poor* object to become *adequate*. Red filled downward-pointing triangles for poor, black filled right-pointing triangles for adequate. Empty rhombuses are outside the domain. The blue dashed line is the 95% confidence limit of the PLSbox inside the latent space.

As we have already pointed out, the inversion of *y*_d_ up to the latent variables space has infinitely many solutions, all obtained when adding points belonging to the null space (Jaeckle and MacGregor, 2000b), precisely, in what we have called the **Q**-null space (Ruiz et al., 2020). Therefore, the set of solutions defined in Eq. 4 is a subspace (a hyperplane) in the latent space, the grey straight line in Figure 6, representing the boundary line for the chosen class-model.

Graphically, all the objects whose scores are “to the right” of the grey line will be inside the class-model of adequate objects. On the contrary, those whose projections are “to the left” of the grey line will be predicted as poor (or, more precisely, they are predicted to be outside the class-model of adequate pellets).

However, it is clear that if the scores move along, for example, the black dotted line (orthogonal to the decision line), eventually, they will fall inside the class-model of the adequate objects. This is the situation illustrated with the different symbols superimposed on the line that starts at one of the poor pellets, the empty square, followed by (computed) scores, red filled downward-pointing triangles, still rejected by the class-model, up to the black filled right-pointing triangles corresponding to points inside the class-model.

Undoubtedly, we can go on moving along the line in the mentioned direction. However, only the valid solutions should be considered, that is, those scores corresponding to objects inside the PLSbox (whose boundary in terms of the 95% confidence level for the *T* ^2^ statistic is depicted as the blue dashed line in Figure 6) and inside the input domain. For example, the three empty rhombuses in Figure 6 follow the right direction, but their corresponding points in the input space, though inside the PLSbox, are outside the domain defined with the range of the variables in the training set, and they should be discarded.

By multiplying by the loading on **P**, as in Eq. 5, the valid scores can be seen in the domain inside the space of the input variables where some of them can be manipulated. The computed solutions are written in Table 1, whose rows follow the order along the direction of improvement in Figure 6. Accordingly, the first three computed objects are rejected by the class-model, the remaining objects are accepted, i.e., inside the class-model of the adequate pellets.

In general, when seeing the computed values in the order of Table 1, in each individual variable, it is shown that to improve the characteristics of the poor object to become adequate the percentage material of all sizes should be reduced as well as the DSC measurements and, at the same time, the TGA and TMA measurements should increase.

Table 1 shows that, following the selected direction from a poor pellet (rejected by the class-model) to an accepted object (inside the class-model) by theoretically modifying its formulation, there is also bounds for these six variables for adequate pellets, namely, Size5 must be less than 12.66, the upper bound of Size10 is 7.55 and 29.49 for Size15, whereas the DSC measurements slowly decrease from 18.60. Similarly, from row four in Table 1, TGA measurements should be greater than 657.69 and TMA measurements start from 55.37. Taking into account the actual domain, defined with the data at hand, the restriction of being in both the PLSbox and the domain also imposes upper bounds for TGA and TMA measurements and lower bounds for the other four variables.

In any case, the variables cannot be varied in the sense of Table 1 independently of each other, they should follow the relation shown in the different rows of Table 1, or any convex combination of any of those rows.

**FIGURE 7:**
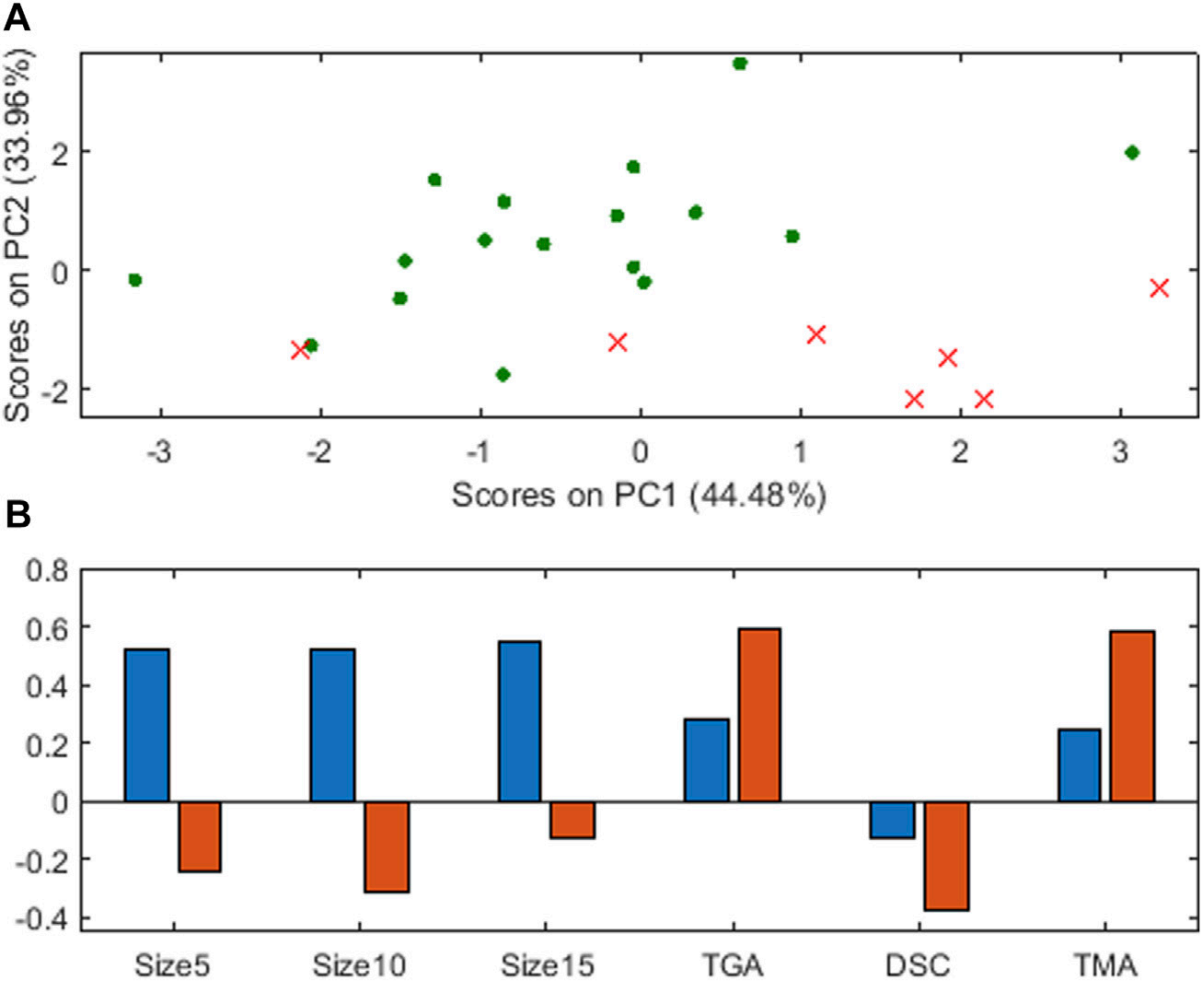
Principal Component Analysis for plastic pellets **(A)** scores on the two first principal components, red crosses for poor, green points for adequate objects **(B)** loadings, blue for the first principal component, orange for the second.

A principal component analysis (PCA) on **X** (autoscaled) shows that the first two principal components, depicted in Figure 7A, also contain information to reasonably distinguish the two classes, in green the adequate pellets and in red crosses the poor ones. It is seen that, qualitatively, to improve the characteristics of the poor objects to become adequate ones is to move in this plane to the left and up, that is, decrease the scores on the first principal component and increase the ones on the second principal component.

Figure 7B shows the loadings on the two principal components, blue for the first, orange for the second. Similar to the previous analysis with Table 1, with the loadings in the first three variables (percentage in the three different size ranges), the *manipulation* should be done clearly decreasing the values of the three variables. The loadings on the last three variables (measurements in different devices) is less clear, but, as the loadings on the second principal component are larger (in absolute value), TGA and TMA should be increased, and DSC decreased.

Nevertheless, questions still remain, such as how much of any one, in which proportion, whether any given relation must be maintained among variables, etc. These questions are answered in the solutions in Table 1, which define the joint combination among all input variables that guarantee a given property.

## Conclusion

PLS-CM models are computed by setting a threshold decision limit in the space of predictions obtained when fitting a binary response that codifies the categories. This limit is selected based on the sensitivity and specificity that are needed in each specific application.

For one of such threshold values, the inversion of the fitted PLS model with a single response defines hyperplanes in both the latent and input spaces that, when observed in the input space, correspond to a kind of prototype of the object belonging to the boundary of the class-model being computed.

For cases where the classes are ‘fail/no fail’, (‘defective/non-defective’) a vector normal to the boundary hyperplane in the latent space defines one direction to move the scores along, exiting the ‘fail’ class to enter the other. In that case, the computed points in the domain corresponding to these scores provide information on how to modify the input variables to improve defective objects. Alternatively, if there is no need of working in the latent space, a direction with the same properties can be obtained directly in the domain by using the boundary hyperplane in the input space.

In that sense, the proposed procedure can be used as a diagnostic tool since it gives the characteristics of the predictor variables (input space) that allow the valid objects to be separated from the invalid ones. The characteristics are precisely those of the objects on the boundary hyperplane of the corresponding class-model. With PLS, contribution plots are common descriptive tools, that allow identification of the variables with the greatest relative influence to discriminate objects of a class in relation to the other. With respect to them, the boundary computed in the latent space with the proposed procedure provides, additionally, estimations of sensitivity and specificity. Furthermore, by “moving” this boundary to the input space, the information about the predictor variables is direct, for example, about how to modify them together pursuing a given goal.

The paper shows some possibilities of acting in specific situations, based on theoretical properties of both the fitted model and its inversion. The theoretical solutions developed in the present work apply in class-modelling contexts, where at least one ‘alternative’ class is adequately represented in the training set together with the target class, and the input variables (at least some of them) can be manipulated. In addition, good predictive PLS models need to be fitted and validated and, whenever possible, the predicted solutions should be experimentally validated.

## Data Availability

Publicly available datasets were analyzed in this study. This data can be found here: http://openmv.net/info/raw-material-characterization http://hdl.handle.net/10259/5753.

## Footnotes

a Available in RIUBU, at http://hdl.handle.net/10259/5753

b Available at http://openmv.net/info/raw-material-characterization
